# Nutrient metabolism and complications of type 2 diabetes mellitus: implications for rehabilitation and precision care

**DOI:** 10.3389/fnut.2025.1699259

**Published:** 2025-10-20

**Authors:** Xue Yu, Xilin Liu, Hong Li

**Affiliations:** ^1^Department of Neurosurgery, China-Japan Union Hospital of Jilin University, Changchun, China; ^2^Department of Hand and Foot Surgery, China-Japan Union Hospital of Jilin University, Changchun, China; ^3^Department of Nursing, China-Japan Union Hospital of Jilin University, Changchun, China

**Keywords:** T2DM, micronutrient deficiency, precision nutrition, glucose monitoring, multidisciplinary rehabilitation

## Abstract

Systemic disruptions in the metabolism of carbohydrates, fats, proteins, and micronutrients cause micro- and macro-vascular damage and impede recovery, which is the driving force behind type 2 diabetes mellitus (T2DM). Neuropathy, nephropathy, foot ulcers, and sarcopenia are symptoms of persistent hyperglycemia, lipotoxicity, excess branched-chain amino acids, and deficiencies in magnesium, zinc, and vitamin D that impair insulin signaling, mitochondrial integrity, and tissue repair. Functional decline is accelerated in skeletal muscle and peripheral nerves due to advanced glycation end-product deposition, ectopic lipid accumulation, and impaired glucose uptake. Micronutrient deficiency hinders wound healing and immune function, while altered nitrogen handling and progressive albuminuria intensify catabolism in the kidney. Controlled protein intake, micronutrient replacement, and microbiome-informed precision diets are targeted nutritional interventions that reduce complications by preserving renal function, restoring nerve integrity, and promoting wound closure. By combining machine learning analytics with continuous glucose monitoring, macronutrient ratios can be changed in real time, improving individualized care. Improved mobility, less neuropathic pain, and better glycaemic control are the results of integrating systematic nutritional assessment and treatment into multidisciplinary rehabilitation protocols. The translation in standard practice continues to be obstructed by inconsistent evaluation tools, lack of availability of omics technologies and few nurse-led randomized trials. Future studies must comprehensively evaluate the long-term effectiveness, cost-effectiveness, and scalability of tailored nutrition in rehabilitation frameworks to lessen the burden of complications and restore functional autonomy in people suffering from type-2 diabetes.

## 1 Introduction

**Figure 1:**
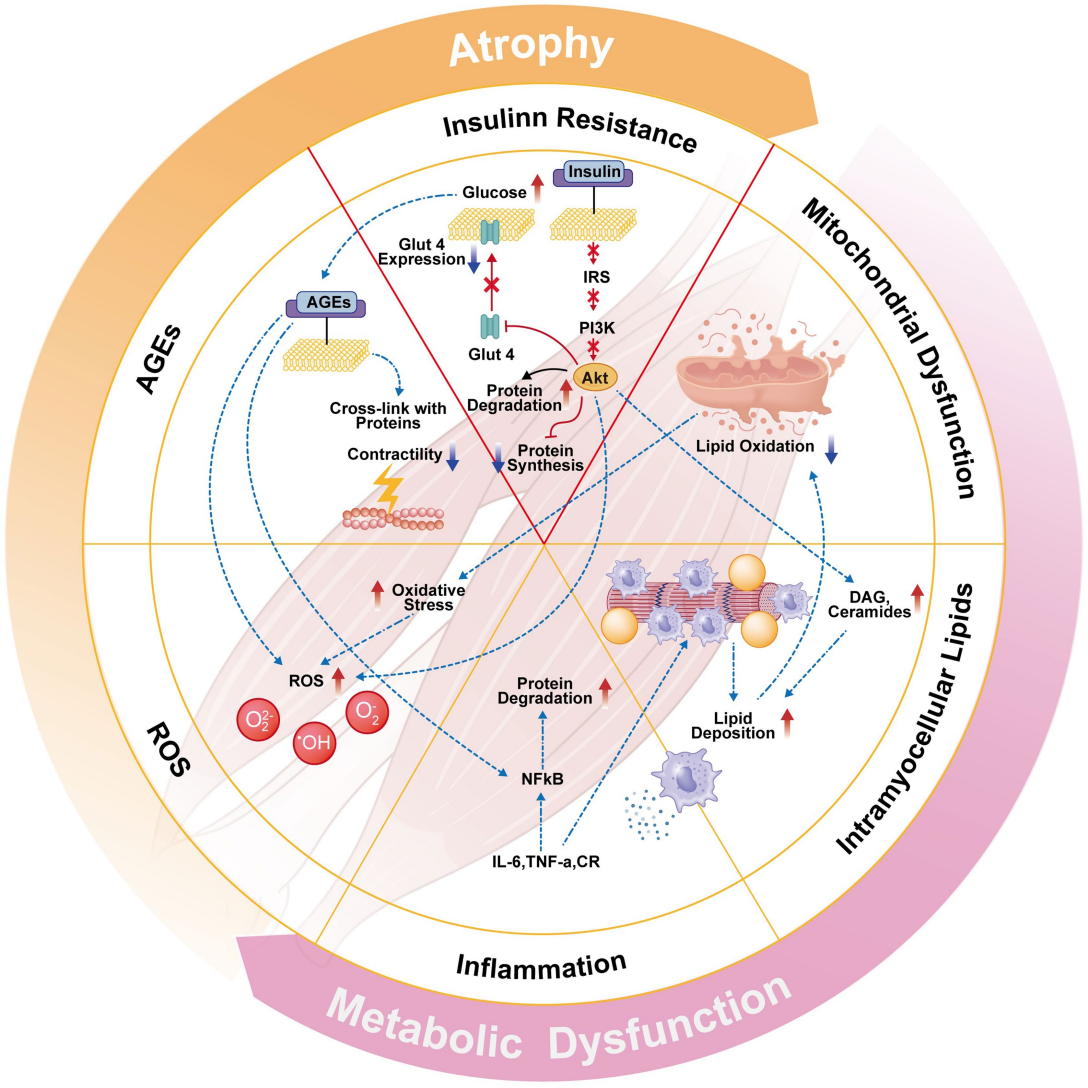
Insulin resistance-induced metabolic dysregulation and its impact on diabetic complications.

The pathophysiological and developmental processes underlying T2DM have important implications for its complications. T2DM induces changes in nutrient metabolism. Insight to undergo precision care and rehabilitation approaches can be gained through studying the latter’s changes. People with type 2 diabetes frequently experience metabolic problems. This includes molecules that interact with diseases and affect the insulin in the body. This leads to complications such as diabetic neuropathy, nephropathy, and musculoskeletal disorders (1, 2). Skeletal muscle is an insulin-sensitive tissue. In type 2 diabetes, skeletal muscle undergoes significant metabolic reprogramming. These include the impairment of amino acid and lipid metabolism, causing insulin resistance and disrupted glucose uptake (1) (Figure 1). On top of exacerbating high blood sugar, this metabolic impairment puts people in danger for complications such as diabetic neuropathy, which at its core involves disturbances in glucose and fatty acid metabolism that lead to abnormal structure and function of peripheral nerves, predisposing them to entrapment neuropathies and irreversible neuronal damage (2).

Emerging evidence suggests that nutrient metabolism contributes to the pathogenesis of diabetic complications, particularly in organs that are sensitive to injury, such as the kidney, retina and peripheral nerve. The role of increased fatty acid and glucose metabolism, mitochondrial dysfunction, and increased protein acetylation in the DKD pathology (2). The energy balance gets disrupted and oxidative stress occurs when glucose metabolism of peripheral nerves is reduced. Conversely, the retina displays a unique metabolic pathway involving the high use of aerobic glycolysis (2). These changes in tissue-specific metabolism underscore the necessity of targeted therapeutics that will help to address the underlying metabolic dysregulation. Moreover, the gut microbiota is vital in regulating nutrient metabolism and interacting with type 2 diabetes complications. Gut dysbiosis in type 2 diabetic patients is characterized by an increased Firmicutes to Bacteroidetes ratio. Moreover, it has been associated with altered bone metabolism and a higher risk of osteoporosis (3). This means that the gut microbiota may be altered with probiotics or dietary changes to treat diabetic bone disorders in the near future. We should add exercise and nutrition to standards of care for the rehabilitation of persons with Type 2 diabetes because of the impact on the metabolism of nutrients, which plays a role in musculoskeletal issues like low back pain and degenerative disk disease (4). Low physical activity is a feature of type 2 diabetes. The condition can worsen metabolic dysregulation and increase the risk of neuromusculoskeletal conditions. It emphasizes the importance of individualized exercise programs to improve glycemic control and reduce disability (4). Lipid Metabolism also plays a significant role in the complications of type 2 diabetes. The disruption of FOXO1-mediated lipid regulation is responsible for the metabolic alterations present in type 2 diabetes mellitus and dyslipidaemic disease (5). Liver disease caused by fatty deposits that are not related to drinking alcohol is increasingly seen as a cause of this condition. There is a strong connection between insulin resistance and the abnormalities associated with type 2 diabetes and the accumulation of fat in the liver, UK-EDM (6). The interventions also aimed at improving metabolic profile. Banit et al. noted in a study that Vertical sleeve gastrectomy improves glucose and lipid metabolism and postpones onset of diabetes in rats. This shows how surgical techniques can have a positive impact on nutrient metabolism (7). Research suggests that L-carnitine may help T2DM patients control blood sugar levels However, a systematic review of L-carnitine supplementation indicates more studies are needed to confirm these results (8). Regulating gut hormones can control type 2 diabetes and help with nutrient metabolism. According to Gribble et al., gut enteroendocrine cells alter their output of hormones in response to the composition of food ingested, the onset of obesity and chemicals secreted by gut micro-organisms. They examine the molecular nature of this process. They also identify certain targets that could be used for treating obesity-associated metabolic disorders like type 2 diabetes (9). Recent studies have also shown that ECM remodeling is crucial for diabetic complications to arise. Focusing on the sarcopenia associated with T2DM, Sun et al. observe that ECM components such as collagen and integrins can facilitate muscle decline through diabetes and may be a promising target for treatment (10).

To make sure people with type 2 diabetes get the right care, we have to completely understand how useful metabolic biomarkers are in predicting complications. Tricarboxylic acid (TCA) cycle intermediates in the urine, which reflect metabolic changes in the diabetic kidney, may be potential prognostic biomarkers for the progression of DKD (2). The use of machine learning methods to predict the metabolite markers of skeletal muscle insulin resistance presents novel opportunities for early diagnosis and targeted therapy (1). These advances in metabolomics and systems biology hold the potential to enable the design of precision care approaches to the metabolic heterogeneity of type 2 diabetes and complications. The consideration of diabetic neuropathy, which has an impact on balance and increase the risk of falling, caused by the factor of nutrient metabolism in type 2 diabetes complications should also be an ideal strategy for rehabilitation (11). Diabetic neuropathy causes dysfunction of the sensory, motor and autonomic nervous systems. Hence, multidisciplinary care comprising physical therapy, nutritional therapy and drugs is required to improve the patient’s outcome (11). In view of the involvement of branched-chain amino acids (BCAAs) metabolism in mitochondrial quality control and cellular ageing, dietary modifications may mitigate the metabolic effects of type 2 diabetes and augment effectiveness of rehabilitation (12). Another key area is the role of the central nervous system in glucose and energy homeostasis As per Sandoval, disturbances in the central nervous system (CNS) fuel sensing pathways controlling glucose and energy homeostasis may contribute to the pathogenesis of type 2 diabetes (13). The progression of the disease may be modulated by therapeutic interventions that target these mechanisms.

The nutritional metabolism has a crucial role in the etiology and developmental complications in type 2 diabetes. It will yield vital information for developing novel rehabilitation and precision care plans. Health care practitioners can improve long-term outcomes in type 2 diabetes and overall patient care by targeting the metabolic dysregulation that underpins the complications of diabetes. Advancements in metabolomics, systems biology, and microbiota are also mentioned. Future studies should aim to clarify the molecular pathways that link nutrient metabolism to issues and the application of these findings for personalized treatments.

## 2 Pathophysiology of nutrient metabolism in T2DM

### 2.1 Perturbations in carbohydrate metabolism

The pathophysiology of nutrition metabolism, especially carbohydrate metabolism, is very closely related to the process of the disease and the development of complications in case of T2DM. Type 2 diabetes is characterized by chronic hyperglycaemia due to peripheral insulin resistance and malfunction of insulin secretion which results in glucose homeostasis dysregulation (14, 15). Alterations of carbohydrate metabolism, where the ability of the body to manipulate and make use of glucose is extensively impaired. In healthy individuals, insulin stops the liver from making glucose and facilitates uptake by other tissues, such as muscle and fat (adipose) tissues. However, in type 2 diabetes, insulin resistance inhibits these mechanisms and glucose rises, especially in fasting and post-prandial states (16, 17). When hyperglycemia lasts for a long time, it not only speeds up the course of type 2 diabetes but also leads to complications. This includes microvascular and macrovascular complications, like diabetic retinopathy, nephropathy, and cardiovascular disease (18, 19).

The impaired inhibition of liver glucose production is characteristic of carbohydrate metabolism diseases in type 2 diabetes. Normally, an insulin helps to suppress the gluconeogenesis and glycogenolysis in the liver. However, in case of type 2 diabetes, the resistance to insulin by the liver causes overproduction of glucose by the liver when the patient is fed (20). Defects in pancreatic *β*-cell function exacerbate this dysregulation, as the compensatory rise in insulin secretion is insufficient to regulate blood glucose levels (21). A vicious cycle is created by the ensuing hyperglycemia because long-term exposure to elevated glucose levels causes glucotoxicity, which further damages *β*-cell function and exacerbates insulin resistance (22). Prolonged hyperglycemia causes advanced glycation end-products (AGEs) to build up, which damages tissue and accelerates the development of diabetic complications (23).

Diets low in carbohydrates have been suggested as a treatment approach for these metabolic disorders. If the diet reduces carbohydrate content, postprandial glycemic load is decreased. This may lessen the insulin secretion demand, leading to better glycemic control (24). Those who support this method cite evidence that carbohydrate restriction produces significant reductions in HbA1c and increases insulin sensitivity (25). According to experts, a carbohydrate-poor diet may lessen your chances of hyperinsulinemia; a condition that is, on its own, associated with insulin resistance and heart problems. This may lessen the post-prandial spikes in both insulin and glucose (26). According to experts, a carbohydrate-poor diet may lessen your chances of hyperinsulinemia; a condition that is, on its own, associated with insulin resistance and heart problems. This may lessen the post-prandial spikes in both insulin and glucose. Recent research has pointed out metabolic markers associated with the progression of type 2 diabetes, which might help you rethink the cause of diabetes other than food changes. A wide range of metabolites have been identified from blood, cerebrospinal fluid, urine, faeces and other biological matrices. Identifying these metabolites is a hot research topic. Metabolomes are used in cell cultures, tissues and organisms (27). According to these findings, metabolic dysregulation encompasses even more serious alterations in nutrient usage besides carbohydrate metabolism, which may aggravate the disease process (28). By identifying these metabolic biomarkers, we can better understand the pathophysiology of type 2 diabetes and identify possible targets for therapeutic intervention, opening the door to more individualized approaches to disease management.

Voluntary exercise imposed during caloric restriction lowers circulating BCAA concentrations, an effect accompanied by amelioration of glucose and lipid homeostasis in experimental models of diabetes mellitus (29). Impaired catabolism of branched-chain amino acids—modulated by pathogenic variants in BCKDHA and loss-of-function alleles in PPM1K—compromises tissue-selective disposal of these metabolites, thereby fostering insulin resistance and accelerating the progression of type 2 diabetes mellitus (30). Blood glucose levels have rapidly returned to normal following interventions like bariatric surgery, indicating that changes in gut hormone signaling and nutrient absorption are crucial. Metabolic surgery reconfigures enteroendocrine signaling and reshapes the gut microbiota, jointly restoring euglycaemia and enhancing whole-body glucose homeostasis (31). Similarly, Roux-en-Y gastric bypass reconfigures bile-acid pool composition and amplifies incretin secretion, effects that converge to attenuate insulin resistance and normalize postprandial glycaemic excursions in diabetic rodents (32). Gut microbiota disturbances are also implicated in nutrient metabolism dysregulation. Shifts in the intestinal microbiota accompany metabolic dysregulation among Japanese individuals with T2DM, modulating nutrient assimilation and systemic metabolic indices (33). This emphasizes how the microbiome affects systemic glucose regulation and carbohydrate metabolism. Ghrelin and incretins are examples of hormone regulators that further alter the metabolism of carbohydrates. Ghrelin engages incretin and insulin signaling cascades, perturbations of which foster insulin resistance in the obese state and thereby presage the onset of T2DM (34). Additionally, Incretin-based therapies potentiate glucose-dependent insulin secretion and refine glycaemic control in individuals with diabetes mellitus (35). Emerging evidence identifies hexokinase domain-containing protein-1 (HKDC1) as a gestational determinant of glucose homeostasis, whose catalytic phosphorylation of glucose modulates maternal glycaemic trajectories. Dysregulated HKDC1 activity during pregnancy precipitates gestational diabetes mellitus, thereby amplifying the lifetime risk of progression to overt type 2 diabetes (36).

Indirect calorimetry, a non-invasive technique, allows for the precise quantification of energy expenditure and respiratory quotient, offering a window into the metabolic shifts that occur in T2DM. Studies have demonstrated that individuals with T2DM exhibit reduced glucose oxidation and increased reliance on lipid metabolism, which may exacerbate insulin resistance and contribute to mitochondrial dysfunction (37). This metabolic reprogramming is particularly evident in skeletal muscle, where impaired glucose uptake and increased intramyocellular lipid accumulation are hallmarks of insulin resistance (38). Indirect calorimetry has revealed that T2DM patients often exhibit elevated resting energy expenditure, which may reflect the heightened metabolic demands associated with chronic hyperglycemia and systemic inflammation (39).

The application of indirect calorimetry in T2DM research has also shed light on the metabolic underpinnings of diabetic complications. In diabetic kidney disease, increased glucose and fatty acid metabolism in the kidney cortex, as measured by indirect calorimetry, has been linked to mitochondrial dysfunction and protein acetylation, which are early events in DKD pathogenesis (24). Similarly, in diabetic neuropathy, impaired glucose metabolism in peripheral nerves, as evidenced by reduced glucose oxidation rates, has been associated with nerve dysfunction and degeneration (25). These findings underscore the utility of indirect calorimetry in identifying tissue-specific metabolic alterations that contribute to the progression of diabetic complications. Moreover, the integration of indirect calorimetry with other metabolomic approaches, such as mass spectrometry and transcriptomics, has enabled the identification of novel biomarkers and therapeutic targets. Increased excretion of TCA cycle intermediates in urine, detected through metabolomic profiling, has been shown to predict DKD progression, highlighting the potential of indirect calorimetry in guiding early intervention strategies (28).

In addition to its diagnostic and prognostic applications, indirect calorimetry has also been instrumental in evaluating the efficacy of therapeutic interventions in T2DM. The use of glucose-dependent insulinotropic polypeptide/glucagon-like peptide 1 receptor (GIPR/GLP-1R) agonists, such as tirzepatide, has been shown to improve glucose and lipid metabolism in T2DM patients, as measured by indirect calorimetry (40, 41). These agents enhance insulin signaling and glucose uptake in adipose tissue, while also promoting lipid clearance, thereby addressing the metabolic derangements that underlie T2DM and its complications (42, 43). Indirect calorimetry has been used to assess the impact of lifestyle interventions, such as dietary modifications and physical activity, on energy expenditure and substrate utilization in T2DM patients. High-fat diets have been shown to exacerbate insulin resistance and impair glucose oxidation, while caloric restriction and ketogenic diets have been associated with improved metabolic flexibility and reduced reliance on lipid metabolism (44).

The metabolism of carbohydrates does not function in isolation in the body. The pathophysiology of type 2 diabetes is further complicated by its effects on lipid and amino acid metabolism. Dyslipidemia causes an increase in triglycerides and a decrease in high-density lipoprotein (HDL) cholesterol which poses a high risk for cardiovascular disease (41). The development of type 2 diabetes and its complications have been linked to disturbances in the metabolism of amino acids, specifically gluconeogenic amino acids (40). These interrelated metabolic pathways demonstrate the complex nature of type 2 diabetes and the necessity of all-encompassing treatment approaches that target the disorder’s wider metabolic dysregulation in addition to carbohydrate metabolism.

### 2.2 Dysregulated lipid metabolism

T2DM and its complications are significantly influenced by disorders of lipid metabolism, especially disorders causing lipotoxicity. The term “lipotoxicity” refers to the consequences of excessive buildup of lipids in the liver, skeletal muscle, pancreas and other non-adipose tissue, causing insulin resistance and failure of the cells (45). High levels of small and dense low-density lipoprotein (LDL) particles, low HDL-C and high triglycerides (TGs) are characteristic of T2DM dyslipidemia, which associates with the disease’s insulin resistance and *β*-cell dysfunction (46). In peripheral tissues, accumulation of lipid metabolites (ceramides and diacylglycerol) interferes with insulin signaling pathways and causes impairment in glucose uptake and metabolism (45). Chronic exposure to elevated free fatty acids (FFAs) leads to *β*-cell apoptosis and decreased insulin secretion, which contributes to the progression of T2DM (45). When patient has Genetically similar to obese nondiabetic rats, diabetic rats develop insulin resistance, impaired fatty acid metabolism, and defective glucose-stimulated insulin secretion (45).

The development of type 2 diabetes like disease have a crosstalk between glucose homeostasis and lipid metabolism disturbance. Increased TGs and FFAs cause not only oxidative stress and systemic inflammation, but also insulin resistance and *β*-cell dysfunction by inhibiting insulin signaling (45). The association between hypertriglyceridemia and diabetic complications including DPN, DR and DKD has been established (47). Individuals with type 2 diabetes and elevated TGs are much more likely to experience these complications, especially if they also have other risk factors like advanced age, prolonged diabetes, and inadequate glycemic control (47). The activation of inflammatory pathways, including c-Jun N-terminal kinase (JNK) and nuclear factor-kappa B (NF-κB), which disturb cellular homeostasis and encourage tissue damage, is one of the molecular mechanisms behind these associations (45).

In people with type 2 diabetes, lipotoxicity also plays a role in the development of macrovascular problems like atherosclerotic cardiovascular disease (ASCVD). By encouraging the production of foam cells and plaque instability, dyslipidemia quickens the course of atherosclerosis (46). Cardiovascular problems arise when oxidized LDL particles build up in the arterial wall because this causes inflammation and endothelial dysfunction (46). Insulin resistance brought on by lipids aggravates dyslipidemia and hypertension, raising the risk of ASCVD even more (46). The role of lipotoxicity in complications of T2DM grandstands the importance of lipid management in diabetes treatment. People diabetes have complications when insulin is not given. It is so essential for the body that we need it even when we are sleeping or resting (46).

Recent studies indicate the importance of the lipid metabolism pathways in preventing and/or ameliorating the complications of type 2 diabetes. Patients affected by type 2 diabetes can benefit from improved lipid profiles and reduced possibilities of cardiovascular events with the use of SGLT2 inhibitors and GLP-1 receptor agonists (24). Strategies that improves the secretion of insulin and sensitivity. It also removes lipids and lowers inflammation helps in managing type 2 diabetes and its complications (24). By developing novel therapies that target specific lipid metabolites, including ceramides and FFAs, we may be able to tackle the root causes of *β*-cell dysfunction and lipotoxicity (45).

The main cause of type 2 diabetes and its complications has been explained as being caused by lipid disorder. Due to the accumulation of toxic lipid byproduct, which disrupts insulin signaling, damages β-cell function, and promote systemic inflammation, there are microvascular and macrovascular complications. The efficient management of lipid metabolism with drugs and lifestyle modifications helps in improving outcome in Type 2 diabetes. Research in the future should focus on the development of directed therapies aimed at the specific mechanisms of lipotoxicity in order to prevent and treat the complications of type 2 diabetes. Multiple studies show that diabetes pathology and lipid metabolism are intimately linked. Khadke et al. made an animal model that replicates human type 2 diabetes. They proved that overexpression of transcription factors, which accelerate hepatic lipogenesis, links to raised hepatic lipid synthesis, which is a TNM2 major characteristic (48). This overexpression implies that the disorder of lipid metabolism linked to type 2 diabetes is significantly influenced by dysregulated lipid synthesis in the liver. Li et al.’s investigation of the function of FOXO1, a transcription factor implicated in lipid metabolism and associated disorders, offers additional understanding of lipid regulation. Their analysis emphasizes how crucial FOXO1 is for regulating lipid homeostasis and suggests that changes in its activity may be a factor in the lipid abnormalities seen in type 2 diabetes (5). Understanding how lipid metabolism is disturbed in diabetic conditions depends on these molecular regulators. Lipid metabolism-focused interventions have demonstrated encouraging results in terms of glycemic control and the advancement of disease. Ileal interposition and vertical sleeve gastrectomy enhance both glycaemic control and lipid handling, deferring the development of diabetes in rodent models. The data indicate that altering the anatomical route and kinetics of nutrient presentation to the distal gut reinstates physiological lipid fluxes and mitigates metabolic dysregulation (7, 49). Bile acid sequestrants have also been demonstrated to control glucose and lipid levels by activating the signaling pathways TGR5 and FXR, which are involved in glucose and lipid homeostasis (50). This mechanism demonstrates how lipid-related pathway modulation may be used therapeutically to control type 2 diabetes. The connection between lipid metabolism and type 2 diabetes is further supported by research on animals. Studies employing rat and hamster paradigms have delineated the molecular circuitry that precipitates lipid dysregulation (48, 51). Through proteomics and metabolomics, Wang et al. specifically discovered abnormalities in amino acid metabolism, suggesting that the metabolic pathways for lipids and amino acids are linked in diabetic pathology (51).

Recent research has thoroughly examined the connection between abnormalities in lipid metabolism and the development of atherosclerosis in people with T2DM. Atherosclerosis in diabetic patients is known to be largely caused by lipid abnormalities, which are defined by changes in the composition of lipoproteins and elevated lipid peroxidation. Insulin-sensitizing agents concurrently enhance glycaemic control and beneficially remodel lipid metabolism together with arterial wall biology, offering a pharmacologic strategy to attenuate atherogenic vascular injury in type 2 diabetes mellitus (52).

One important element in atherogenesis has been found to be lipid peroxidation in low-density lipoprotein (LDL) particles. Lankin et al. measured lipid peroxide content in plasma LDL from patients with various cardiovascular and metabolic conditions, including T2DM (53). The results highlight the correlation between elevated lipid peroxides in LDL and both diabetes and cardiovascular complications, suggesting that oxidative lipid modification is a major factor in the development of atherosclerosis in diabetics. Thiazolidinediones and other antidiabetic medications have been shown to have anti-inflammatory and anti-atherosclerotic effects. These compounds attenuate vascular inflammation, an action that underlies their ability to oppose atherogenesis in the context of type 2 diabetes mellitus (54). Similarly, Gautam et al. also clarified the function of macrophage receptors, specifically CD36, in mediating oxidized LDL (Ox-LDL) uptake. They found a correlation between Ox-LDL receptor activity and type 2 diabetes, highlighting the significance of lipid uptake mechanisms in diabetic atherosclerosis (55). Lipid and carbohydrate metabolic disorders in the context of cardiovascular disease are also influenced by adipokines and hormone regulators. A study by Shibata et al. investigated the pathogenic mechanisms of hormones in adipose tissue, which are linked to lipid dysregulation and the development of atherosclerosis (56). Kvandová and colleagues investigated the function of PPARγ, a nuclear receptor implicated in the regulation of lipid metabolism, emphasizing its importance in cardiovascular disorders linked to type 2 diabetes (57). Pharmacological treatments that target inflammation and lipid metabolism have been the subject of recent experimental investigations. According to Nakatsu et al., SGLT2 inhibitors, like luseogliflozin, suppress the development of atherosclerosis in diabetic mouse models and quickly restore inflammation-related gene expression in the aorta. This suggests that altering lipid-related inflammatory pathways may have an impact on the progression of atherosclerosis (58). Metabolomic techniques have shed light on serum biomarkers linked to the development and complications of type 2 diabetes. To determine serum metabolite profiles that differentiate non-diabetic people, treatment-naïve T2DM patients, and those with complications, Coco et al. used NMR-based metabolomics. This revealed metabolic abnormalities connected to dysregulation of lipid metabolism (16). Diabetic complications, including diabetic nephropathy, have been linked to lipid accumulation and degradation pathways, including lipophagy. Yang et al. suggested lipophagy as a possible therapeutic target to address lipid-related pathology in diabetics and talked about how abnormalities in lipophagy contribute to ectopic lipid deposition in renal tissues, promoting disease progression (59).

### 2.3 Protein and amino-acid metabolism

Lipid profile changes, such as increased triglycerides (TAG), low-density lipoprotein-cholesterol (LDL-C), and decreased high-density lipoprotein-cholesterol (HDL-C), are indicative of the metabolic dysregulation seen in type 2 diabetes and are strongly associated with insulin resistance and hyperglycemia (46, 60). The pathophysiology of cardiovascular diseases (CVD), a major cause of morbidity and death in individuals with type 2 diabetes, is influenced by these lipid abnormalities (46, 60). Furthermore, it is becoming more widely acknowledged that a key contributing factor to diabetic complications is the interaction between lipid metabolism and protein/amino acid metabolism. Insulin resistance and the advancement of type 2 diabetes have been linked to BCAAs and aromatic amino acids, whose metabolism is severely impaired in patients with more severe complications (16, 41). In addition to making insulin resistance worse, the dysregulation of these metabolic pathways causes harmful metabolites like AGEs to build up and further harm organs and tissues (2, 61).

Certain serum and plasma metabolites have been linked in recent metabolomics studies to complications of type 2 diabetes, such as diabetic polyneuropathy (DPN), diabetic retinopathy (DR), and DKD (19, 23). DPN has been associated with changes in acylcarnitines, sphingolipids, and free fatty acids, indicating a new function for lipid metabolism in nerve injury (23). Alterations in the metabolism of amino acids, specifically the build-up of TCA cycle intermediates in urine, have been suggested as prognostic biomarkers for the advancement of DKD (2, 41). Due to the different metabolic reactions of the kidney, retina, and peripheral nerve to hyperglycemia and lipid dysregulation, these findings highlight the significance of comprehending the tissue-specific metabolic reprogramming in type 2 diabetes (2).

The complications of type 2 diabetes is further complicated by the role post-translational modifications (PTMs) play in nutrient metabolism. T2DM modifies the glycosylation and phosphorylation of serum proteins, affecting the bioactivity of metabolic regulators such as ERK and insulin (2, 61). These changes may serve as possible therapeutic targets as well as being implicated in the pathophysiology of complications. Targeting the hepatic secretome or hepatocyte nuclear factors (HNFs) has shown to modulate lipid and glucose metabolism and reduce the complication risk of diabetes (61). Moreover, preclinical models show that treatments capable of reinstating mitochondrial function and lowering oxidative stress, such as nicotinamide N-methyltransferase (Nnmt), have metabolic advantage (26).

Combining metabolomics with clinical data may completely change how we treat complications of T2DM. Through the identification of disease-associated metabolic fingerprints, doctors can predict the likelihood of downstream complications and tailor their interventions (2, 16). Through the application of 1H-NMR-based metabolomics, distinct metabolic differences in T2DM patients with and without complications have led to the development of composite risk scores (16). A non-invasive way to track the course of a disease and assess the effectiveness of treatment is to identify biomarkers, such as urinary TCA cycle intermediates (2).

With new research pointing to changes in amino acid handling and metabolic regulation, the pathophysiology of T2DM is significantly influenced by the metabolism of proteins and amino acids. Recent studies have started to clarify the intricate interactions involving amino acids in type 2 diabetes, whereas a large portion of the early research concentrated on insulin resistance in glucose and lipid pathways. Insulin resistance in diabetes was first studied, and it was found that type 1 diabetes involves resistance to amino acid metabolism in addition to glucose. It was shown by Tessari et al. that people with insulin-dependent diabetes are resistant to the suppressive effects of insulin on leucine oxidation and appearance, suggesting a more widespread impairment in amino acid metabolism linked to insulin resistance (62). According to Inchiostro et al., type 1 diabetes also causes an increase in endogenous amino acid turnover, especially for essential amino acids like leucine, both at baseline and during hyperinsulinemic and hyperaminoacidemic episodes (63). Interestingly, leucine’s ability to be used for protein synthesis was unaffected, indicating unique changes in amino acid kinetics rather than a general deficiency. It has been investigated whether insulin resistance has a comparable impact on protein and amino acid metabolism in the setting of type 2 diabetes. Insulin resistance in type 2 diabetes is mainly linked to lipid and glucose pathways, but it is unclear how it affects the metabolism of proteins and amino acids (64). Although some evidence suggests variability, particularly in obese male patients, the majority of studies show that the stimulation of whole-body protein synthesis during hyperinsulinemia appears to be normal in T2DM subjects (64). The therapeutic potential of amino acid-based nutritional interventions has also been studied. In older patients with poorly managed type 2 diabetes, Solerte et al. assessed the metabolic effects of oral amino acid supplementation, indicating that amino acid intake may affect metabolic parameters (65). Aquilani discussed the potential of oral amino acid administration as either a supplement or a metabolic therapy in diabetes management, emphasizing the importance of amino acids in modulating metabolic homeostasis (66). Coco et al. employed NMR-based metabolomics to identify serum biomarkers associated with T2DM progression, revealing alterations in amino acid profiles that correlate with disease development and complications (16). The potential of amino acid metabolites as markers of disease state and progression is highlighted by these findings. Hu et al. has helped in a mechanistic understanding of amino-acid sensing and its metabolic regulation by describing how amino-acid sensing mechanisms respond to the availability of amino acids and modulate the metabolism of energy, glucose and lipids (67). Disruption of this network critical for maintenance metabolic homeostasis could actually contribute to pathophysiology of type 2 diabetes.

### 2.4 Micronutrient deficiency and the pathogenesis of T2DM complications

Due to high diabetes prevalence across the globe and its association with microvascular and macrovascular complications, it is important to study the relationship between micronutrient deficiency and T2DM complications. Deficiencies in micronutrients, including zinc, potassium, calcium, magnesium, and vitamin D, significantly increase the risk of poor glycemic control and onset of diabetic complications. These elements are vital for insulin production, glucose metabolism, and metabolic signaling (68, 69). Magnesium is a cofactor for various enzymes involved in glucose and insulin activity, and zinc is required for the biosynthesis of insulin and receptor sensitivity (17, 68). Low levels of these micronutrients are associated with high glycated hemoglobin (HbA1c), a marker of long-term metabolic control, and increased risk of complications such as diabetic retinopathy (DR), diabetic nephropathy (DN) and DPN (70, 71).

Recent studies have shown that individuals with diabetes may reduce their HbA1c level and gain control of the disease merely by increasing their intake of potassium and magnesium. In addition, an independent link has been shown between vitamin D deficiency and an increased risk of the complications known as DPN and DN. Furthermore, lower serum 25-hydroxyvitamin D (25 (OH)D) levels were reported to correlate with increased prevalence and severity of DPN and DN (70, 72). Since oxidative stress, inflammation, and immune responses are important pathways in the pathophysiology of diabetic complications, micronutrients play a role that goes beyond glycemic control (73, 74). Vitamin D reduces inflammation and insulin resistance, while zinc and magnesium are part of antioxidant defense systems (74, 75).

A major contributing factor to complications from diabetes is vitamin D deficiency. According to Jung et al., serum 25 (OH) D levels below 10 ng/mL are independently linked to a higher risk of DN in females with type 2 diabetes and DPN in males (76). Maintaining appropriate vitamin D levels is crucial to preventing severe limb outcomes, as Qian et al. found that 25(OH) D deficiency is a critical risk factor for minor amputations in patients with diabetic foot ulcers (77). These results imply that the pathophysiology of microvascular complications in type 2 diabetes may be influenced by vitamin D deficiency.

Beyond vitamin D, other micronutrients such as zinc and vitamin B12 are also implicated. Al-Komi and colleagues documented zinc depletion in post-bariatric individuals, a cohort already predisposed to micronutrient deficits; its contribution to the progression of diabetic complications remains to be rigorously elucidated (78). Sayedali et al. identified a high prevalence of vitamin B12 insufficiency among metformin-treated individuals with type 2 diabetes, underscoring the drug’s intrinsic propensity to impair cobalamin absorption and the consequent need for systematic surveillance (79). They emphasized that the use of metformin can prevent the absorption of B12, resulting in a deficiency that can have negative clinical consequences, such as anemia and neurological problems.

Iron deficiency anemia (IDA) is another micronutrient-related concern in T2DM. Iron deficiency may worsen diabetic complications by increasing oxidative stress and impairing oxygen delivery, according to a meta-analysis showing that patients with IDA treated with metformin have noticeably lower hemoglobin levels (80).

Micronutrient deficiencies are common among bariatric surgery candidates and a high prevalence of different deficiencies, such as those in vitamins and minerals, was observed in this population (81). Such deficiencies could predispose individuals to further complications if not properly managed.

Patients with type 2 diabetes have a startlingly high prevalence of micronutrient deficiencies, especially in areas like South Asia and sub-Saharan Africa where access to a variety of nutrient-dense diets is restricted (69, 82). Micronutrient deficiencies affect insulin signaling and glucose metabolism, which speeds up the onset and progression of complications, making this “hidden hunger” worsen the burden of diabetes (69, 83). The potential of micronutrient supplementation as a therapeutic approach has been highlighted by systematic reviews and meta-analyses, which have underlined the necessity for thorough research to evaluate the burden of multiple micronutrient deficiencies and their effect on glycemic control (69, 84). A sustainable way to address deficiencies in at-risk populations is through innovative methods like biofortifying staple crops with vital micronutrients (82). Personalized treatment plans could be improved by incorporating micronutrient assessment into routine diabetes care, especially for patients with advanced complications or poor glycemic control (68, 85). Since some genetic variations may increase susceptibility to deficiencies and their negative effects, the discovery of genetic polymorphisms linked to micronutrient metabolism and diabetic complications emphasizes the significance of customized interventions even more (74, 86).

## 3 Nutrient-related complications and their impact on rehabilitation

### 3.1 Diabetic peripheral neuropathy (DPN)

A common and crippling side effect of T2DM, DPN affects roughly 50% of people with the disease and severely lowers quality of life (87, 88). The progressive damage of peripheral nerves is the main component of this disease and causes pain, sensory loss and changes of mechanosensitivity. Abnormal gait, balance issues, falls, and diabetic foot ulcers can result from these symptoms (89, 90). DPN is a result of several factors, including oxidative stress, sustained high blood sugar levels over time (chronic hyperglycemia), as well as the mechanics of the nerve (91, 92). There are no disease-modifying therapies available for DPN due to its high prevalence. Early recognition and preventive measures play an important role in DPN management (93).

Research on the association of DPN and the nutrient-related complication of T2DM has received a lot of research interest due to its impact on the quality of life and rehabilitation strategies of the patients. Research shows that the majority of patients do not recognize their neuropathic symptoms. This often leads to a missed diagnosis and delay in treatment of DPN, a common and serious complication of type 2 diabetes (94). The high incidence of DPN in Korean populations highlights the clinical significance of the condition and the necessity of efficient screening and intervention strategies (95).

Although other metabolic factors, such as components of the metabolic syndrome (MetS), may also play a role, research indicates that hyperglycemia, a hallmark of type 2 diabetes, is a key mediator in the pathogenesis of DPN. Although hyperglycemia is a significant factor, Grisold et al. point out that the role of MetS and its constituent parts needs more research because they might be additional causative factors in the development of neuropathy (96). Nonmetabolic factors, including genetic susceptibility, age, sex, smoking, and alcohol consumption, have also been identified as potential risk factors, although their precise contributions remain unclear (96). Variability is revealed by prevalence studies across various populations, in part because of variations in study populations and diagnostic standards. Particular risk factors for DPN were found in a study conducted in rural North India, highlighting the impact of regional lifestyle and demographic factors on disease burden (97). Diabetic patients with peripheral neuropathy have significantly worse everyday functioning and well-being, according to a pilot study assessing the effect of DPN on quality of life (98).

Complex pathophysiological mechanisms underlie DPN, and AGEs are being studied as possible causes. AGE accumulation and the degree of neuropathy were not directly correlated in Singh et al.’s analysis of AGE levels in T2DM patients, indicating that alternative pathways might be at play in the development of DPN (99). This suggests that although metabolic disorders and hyperglycemia are major, their aftereffects are complex.

Clarifying DPN mechanisms has been made possible in large part by animal models, with mouse models offering valuable information on the neurologic phenotypes linked to type 2 diabetes. In their discussion of the usefulness of different models for researching DPN, O’Brien et al. stress the significance of choosing the right models to comprehend the course of the disease and evaluate possible treatments (100). Predictive analytics developments like machine learning are becoming useful instruments for DPN early detection. A machine learning-based model that can forecast DPN risk was created and validated by Sun et al.; this model has the potential to enhance preventive measures and customize rehabilitation initiatives (101). Interventions that focused on enhancing balance and coordination, which are essential for rehabilitation, showed that training with the Biodex Balance System can improve DPN patients’ static balance and coordination, underscoring the possibility of using targeted physical therapy to lessen functional impairments (102).

The development of DPN is accelerated and rehabilitation attempts are complicated by nutrient-related complications in type 2 diabetes, especially deficiencies in vital vitamins and minerals. Due to its critical role in nerve health and insulin secretion, vitamin D deficiency has been associated with an increase in neuropathic symptoms. Research has demonstrated that combining physical exercise with vitamin D supplements can greatly reduce the symptoms of sensory-motor neuropathy, such as tingling, pain, and numbness, while also improving tactile sensitivity and vibration perception in DPN patients (103). This demonstrates how combining physical rehabilitation with nutritional interventions may help lessen the negative effects of DPN and enhance patient outcomes.

Restoring nerve function and increasing mobility through neurodynamic techniques (NDT) and neural mobilization (NM) are common goals of rehabilitation strategies for DPN. IIt has been suggested that NDT, like tibial nerve mobilization, improves nerve conduction parameters, reduces neuropathic pain and improves the quality of life in DPN patients (104). The use of NM techniques which target intraneural and extraneural effects can decrease the risk of diabetic foot ulcers. Furthermore, NM techniques have been shown to restore normal physiological nerve function. It can also improve ankle mobility and the distribution of plantar pressure (105, 106). A multidisciplinary approach essentially a combination of physical therapy and pharmacotherapy appears to be the most appropriate way to rehabilitate patients with neuropathy. One of the novel technologies, diffusion tensor imaging (DTI), offers new tools for evaluating peripheral nerve integrity and monitoring the effects of treatment in DPN. The DTI parameters, ADC and FA, are believed to provide microstructural information on peripheral nerve damage in hyperglycemia, and may be indicative of the clinical utility to assess outcome following NM techniques (107). This is a significant breakthrough since it allows objective and non-invasive assessment of nerve condition, the action of nerves, and the outcome of treatment. It will allow personalized rehabilitation programs to be formed.

The treatment of DPN seems promising with the help of dietary changes and supervised exercise training as two lifestyle interventions. Intensive lifestyle interventions (dietary counseling, physical activity, and actigraphy-based behavioral changes) are associated with better neuropathy progression outcomes and quality of life in DPN patients. The management of DPN can benefit from a multidisciplinary approach. These interventions not just regulate the metabolism that is going wrong, they also facilitate regeneration of the peripheral nerve as well as functional recovery.

### 3.2 Diabetic kidney disease

DKD, a common consequence of type 2 diabetes, is a severe issue that significantly strains health systems around the world. This is due to its association with end-stage kidney disease (ESKD) as well as increased cardiovascular morbidity and mortality (108, 109). Besides other risk factors, such as hypertension, hyperlipidemia, and diabetic retinopathy, structural and functional alterations in the kidneys induced by hyperglycemia are part of the DKD multifactorial pathophysiology (110, 111). Seventeen to 26 % of patients with T2DM have albuminuria, suggesting early kidney injury. This indicates the importance of early detection and management (112). The KDIGO guidelines recommend assessing estimated glomerular filtration rate (eGFR) and albuminuria together to assess the risk of kidney disease and direct management (110, 113). The rate of DKD has not changed dramatically, although blood pressure and glycemic control continue to improve, indicating a need for new therapies (112).

The links between nonalcoholic fatty liver disease (NAFLD), a new cardiometabolic risk factor, and the development type 2 diabetes and its complications are described (114). This association highlights the importance of metabolic health in the mitigation of diabetes complications. In addition, certain diets like Mediterranean, ketogenic, DASH, and vegetarian have been shown to slow the progression of DKD. This highlights the importance of dietary interventions (115) (Table 1).

**Table 1:** Dietary interventions modulate gut microbiota.

Dietary factors	Microbiota modulation	Metabolism	Refs.
Pumpkin polysaccharide	Modulate the gut microbial composition to selectively enrich key taxa such as Bacteroidetes and Prevotella	Ameliorate insulin resistance and reduce fasting glucose, total cholesterol, and low-density lipoprotein cholesterol levels while concomitantly elevating high-density lipoprotein cholesterol levels	(174)
DNA-guided precision nutrition	N/A	Lower fasting plasma glucose and glycated hemoglobin levels, potentially retarding the progression of type 2 diabetes mellitus	(175)
Lessonia nigrescens ethanolic extract	A compositional shift toward higher Bacteroidetes, lower Firmicutes, and selective expansion of Barnesiella—a genus consistently linked to metabolic benefit	Euglycaemic restoration, preservation of hepatic and renal cytoarchitecture, and amelioration of insulin resistance via selective modulation of the PI3K and JNK signaling cascades	(176)
*Hylocereus polyrhizus* fruit betacyanins	Change the composition of gut microbiota and increase the relative abundance of Akkermansia	Mitigating diet-induced obesity, hepatic steatosis, and insulin resistance while ameliorating the inflammatory status	(177)
Combination of aronia, red ginseng, shiitake mushroom, and nattokinase	Preventing diabetes-induced alterations in gut microbiota composition	Enhancing insulin secretion, reducing insulin resistance, improving glucose metabolism, and preserving bone mineral density	(178)
Green-Mediterranean diet: plant-enriched, walnuts, Mankai, green-tea polyphenols; restricted red and processed meat; isocaloric	↑ Prevotella, ↑ BCAA-catabolizing pathways; ↓ Bifidobacterium, ↓ BCAA-biosynthetic modules; shifts driven by low-abundance “non-core” taxa	Stepwise reductions: body-weight (−6.5%), waist circumference, HOMA-IR, Framingham risk; 12% of weight-loss and 18% of risk-score reduction statistically mediated by microbiome compositional change	(179)
Long-term Mediterranean diet adherence	↑ *Faecalibacterium prausnitzii*, *Eubacterium eligens*, *Bacteroides cellulosilyticus*; ↓ *Ruminococcus torques*, *Collinsella aerofaciens*. Protective association amplified in individuals with low/absent *Prevotella copri*	Lower cardiometabolic risk score (composite of HbA1c, lipids, hs-CRP); 18% lower predicted MI risk per 4-unit MedDiet increment in *P. copri*–negative participants	(180)
Two-week whole-food, high-fiber intervention	↑ Bifidobacterium, Lactobacillus, Prevotella; community *β*-diversity shifted within subjects (R^2^ = 8.3%); α-diversity decreased; no strain-level change in *Eubacterium rectale*	↑ inositol-degradation gene abundance; fecal SCFAs (acetate, propionate, butyrate) unchanged; functional CAZy repertoire stable	(181)
Pomegranate extract vs. placebo in polyphenol-naïve, poly-medicated MetS patients	↑ Lactococcus (AD, LL, HP groups); ↑ Bifidobacterium (AD, LL only); ↓ Clostridium XIVa (non-LL, non-HP); drug therapy modulates magnitude and direction of shifts; no global α- or β-diversity change	↓ plasma lipopolysaccharide-binding protein (LBP) in all patients; ↓ soluble ICAM-1 in LL-treated subgroup; circulating SCFAs unchanged; urolithin metabotypes & SNPs did not influence response	(182)
Mediterranean, high-fiber, polyphenol-rich patterns; avoidance of western diet	↑ SCFA-producing commensals (Faecalibacterium, Roseburia, *Akkermansia muciniphila*); ↓ endotoxin-producers (Escherichia, Streptococcus, Dorea); restoration of dysbiosis	↑ intestinal SCFAs → enhanced barrier, ↓ LPS translocation, ↓ TMAO, improved hepatic lipid oxidation, reduced steatosis & fibrosis; AI-guided personalization improves adherence and metabolic outcomes	(183)
Short-term dietary reduction of BCAAs attenuates postprandial insulin secretion and ameliorates white adipose tissue metabolism and gut microbiota composition in individuals with type 2 diabetes	Dietary BCAA restriction is associated with alterations in gut microbiota composition, specifically an increased abundance of Bacteroidetes and a decreased abundance of Firmicutes following reduced BCAA intake	Short-term dietary intervention attenuates postprandial insulin secretion without improving systemic insulin sensitivity in patients with type 2 diabetes	(184)
A low-calorie formula diet, followed by a food reintroduction and weight-stabilization phase, markedly reshapes the gut microbiota composition in obese adults with type 2 diabetes	Following the low-calorie formula diet, both microbial richness and the Shannon diversity index increased, while the genus Collinsella showed a marked decrease in relative abundance	The weight-loss program improved glycaemic control, as evidenced by significant reductions in HbA1c, fasting plasma glucose, and insulin concentrations	(185)
The fiber-rich Ma-Pi 2 diet significantly ameliorates metabolic control in patients with type 2 diabetes	The Ma-Pi 2 diet markedly augmented beneficial SCFA producers and Akkermansia-while concomitantly reducing potentially pro-inflammatory taxa	The Ma-Pi 2 diet improved glycaemic control, as evidenced by significant reductions in HbA1c, fasting plasma glucose, insulin resistance, total cholesterol, and body weight	(186)
A high-fiber diet enhances glycaemic control in individuals with type 2 diabetes by selectively promoting specific SCFA-producing microbial taxa	The high-fiber diet markedly increased the abundance of 15 SCFA-producing bacterial taxa that play pivotal roles in improving host metabolic health	A high-fiber diet improves glycaemic control by lowering HbA1c levels and potentiating insulin secretion via enhanced GLP-1 production	(187)
Dietary fiber is fermented by the gut microbiota to produce SCFAs	SCFAs modulate host physiology by activating GPCRs and inhibiting HDACs	SCFAs exert pleiotropic metabolic effects on the host, including enhancing insulin sensitivity, modulating energy metabolism, and attenuating inflammation	(188)
Effects of a prebiotic mixture on gut microbiota composition, gastrointestinal symptoms, energy levels, and perceived hunger in healthy adults	The prebiotic mixture markedly reshaped gut microbiota composition, enriching beneficial taxa linked to cardiovascular health while depleting those associated with adverse health outcomes	The prebiotic mixture ameliorated gastrointestinal symptoms, increased perceived energy, and reduced hunger, accompanied by significantly enhanced postprandial satiety and energy levels	(189)
The Mediterranean diet reduced plasma cholesterol and modulated the gut microbiota in overweight and obese subjects	Mediterranean-diet intervention induced gut-microbiota shifts, increased gene richness, and was observed specifically in individuals who exhibited a concurrent reduction in systemic inflammation	Following Mediterranean-diet intervention, subjects exhibited elevated urinary urolithin levels and enhanced fecal bile-acid degradation, both of which co-varied with specific microbial taxa	(190)
This study investigated the impact of dietary location on the gut microbiota under conditions of IBD	CD patients display location-specific dysbiosis, most notably a deficit in SCFA biosynthetic pathways	Functional prediction revealed altered microbial functional potential across multiple disease locations in CD patients, with a significant reduction in SCFA-producing pathways compared to healthy controls	(191)
A multifunctional dietary intervention attenuated intestinal inflammation in individuals with cardiometabolic risk	The MF intervention increased the relative abundance of beneficial bacteria, while decreasing the abundance of *Akkermansia muciniphila*	The MF intervention reduced serum levels of BCAAs and glutamate, and these changes were associated with improved intestinal inflammation	(192)
The water-soluble tomato extract rich in secondary plant metabolites (Fruitflow) reduced TMAO levels	Fruitflow intervention altered the β-diversity of the gut microbiota, specifically increasing the relative abundance of Alistipes while decreasing the abundances of Bacteroides and Ruminococcus	Fruitflow intervention reduced plasma and urinary TMAO levels and concurrently decreased plasma endotoxin concentrations	(193)
A Mediterranean diet intervention modulated the gut microbiota composition in older adults	Mediterranean-diet intervention enriched health-associated taxa such as Faecalibacterium, prausnitzii, while depleting frailty-related microbes	Mediterranean-diet intervention was associated with increased levels of SCFAs and BCFAs, alongside reduced secondary bile acid	(194)

PI3K, phosphatidylinositol 3-kinase; JNK, c-Jun N-terminal kinase; SCFAs, short-chain fatty acids; BCFA, branched-chain fatty acids; TMAO, trimethylamine N-oxide; LL, lipid-lowering; HP-, anti-hypertensive (−); AD-, anti-diabetic; LPS, lipopo-lysaccharide; BCAA, branched chain amino acid; MF, multifunctional dietary; CD, Crohn’s disease; IBD, inflammatory bowel disease; GPCRs, G-protein–coupled receptors; HDACs, histone deacetylases.

Many different processes are involved in oxidative stress, the autophagy pathway, and inflammation of DKD pathophysiology. As a major treatment for T2DM, metformin has been found to activate Sirt1/FoxO1 autophagic signaling so as to reduce renal damage, thereby indicating that modulation of autophagy pathways may help to prevent and/or delay DKD (116). Pharmacological management of nutrient-related metabolic disturbances is crucial, as evidenced by the effectiveness of SGLT2 inhibitors such as canagliflozin in lowering the risk of kidney failure and cardiovascular events in patients with type 2 diabetes and established nephropathy (117, 118).

Novel treatments like mesenchymal stem cell (MSC) therapy have the potential to reduce chronic inflammation and enhance long-term diabetic complications, such as nephropathy (119). These methods imply that addressing metabolic dysregulation and inflammation at the cellular level may be a practical way to treat renal complications brought on by nutrients. For early detection and treatment, the clinical assessment of microvascular complications, such as nephropathy, is still essential. Microalbuminuria is common in type 2 diabetes patients. It is often regarded as an early indicator of renal damage. Also, it is linked to other diabetic complications such as hypertension (120, 121). The discovery of microalbuminuria as an alert marker emphasizes the need for proper metabolic management and dietary control to prevent the disease’s progression. In recent research articles, new drugs such as GLP-1 (glucagon-like peptide-1 receptor agonists), SGLT2 (sodium-glucose cotransporter-2 inhibitors) and RAAS (renin-angiotensin-aldosterone system inhibitors) have been found to slow down the progression of DKD (110, 111). Despite their glucose-lowering mechanisms, these substances also have renoprotective and cardioprotective effects in addition to improving glycemic control (105, 106). In patients with and without type 2 diabetes, SGLT2 inhibitors like canagliflozin and dapagliflozin have been demonstrated to dramatically lower the risk of adverse kidney outcomes. GLP-1 RAs, such as semaglutide, have also shown promise in real-world clinical settings for postponing microvascular and macrovascular complications, including DKD (110). These agents are also known to possess renoprotective and cardioprotective activities in addition to their glucose-lowering effects (94, 95). SGLT2 inhibitors such as canagliflozin and dapagliflozin have been shown in patients with and without type 2 diabetes to significantly reduce adverse kidney outcomes. GLP-1 RAs, such as semaglutide, have also shown promise in delaying microvascular and macrovascular complications, including DKD, in a real-world clinical setting (112). Multidisciplinary collaboration with endocrinologists, nephrologists, cardiologists, and primary care doctors is important to optimize outcomes (122). According to a new study in collaboration with the University of Bristol, it was revealed that obesity can change a person’s genetic makeup (122). Engaging a Certified Diabetes Care and Education Specialist (CDCES) in the treatment team can further improve long term outcomes and adherence to treatment (122).

Emerging evidence also highlights the importance of nutritional interventions to reduce DKD-related complications due to nutrients. Malnutrition, vitamin deficiencies, and dietary changes are frequent issues for T2DM patients with chronic kidney disease (CKD), requiring specialized nutritional approaches (105). People with advanced DKD often lack niacin and fat-soluble vitamins, particularly those given somatostatin analog treatment. Through dietary counseling and supplementation of the nutritional deficiencies found from the blood test, patients’ overall health may be improved and rehabilitation can be aided (105). By treating these nutritional deficiencies with dietary counseling and supplements, patients’ general health can be enhanced and rehabilitation efforts can be supported (105). To prevent the progression of CKD in individuals with T2DM, modifiable risk factors such as hyperlipidemia, hypertension and inactivity should be identified and treated (110, 111).

### 3.3 Recalcitrant wound healing

The development and management of diabetic foot ulcers (DFUs) and chronic wound healing are largely related to nutrient-related complications of T2DM, making them important rehabilitation strategies. One of the most serious consequences of diabetes is diabetic foot. It is associated with ulceration due to peripheral arterial disease and/or neuropathy. In rare cases, lower limb amputation and chronic non-healing ulcers may occur due to these conditions (123). Wound healing is made more difficult by the inherent pathophysiological abnormalities in type 2 diabetes, including decreased angiogenesis, increased oxidative stress, and aberrant inflammatory responses (106). Nutritional deficiencies, which are crucial in postponing wound healing and raising morbidity, exacerbate these factors even more (123). Nutrition has a well-established role in wound healing; cellular metabolism, immunological response, and tissue repair all depend on macronutrients like protein and micronutrients like vitamins and trace elements. Important nutrients for wound healing, such as albumin, hemoglobin, iron, and zinc, are frequently deficient in diabetic patients (123). While iron and zinc deficiencies affect collagen synthesis and immune response, respectively, low serum levels of albumin and hemoglobin have been linked to poor wound healing rates (123). The need for energy and nutrients is increased by the hypermetabolic state of chronic wounds, but diabetic patients are frequently prescribed restrictive diets to control their blood sugar levels, which results in insufficient nutrient intake.

Recent studies show how nutrient supplements can help diabetic patients with quicker wound healing. According to a study, taking zinc supplements improves collagen production. Collagen production and immune function are essential for wound healing (123). NHowever, because nutrition assessment is not often included in standard care protocols for DFU, these findings remain limited in their clinical application (123). The gap in practice points to the need for a multi-disciplinary approach that includes nutritional assessment and supplementation in comprehensive wound care plans. The multimodal team member approach which includes Wound care specialists, endocrinologist, dietitian, and rehabilitation specialist have been shown to be the best strategy for the treatment of diabetic foot disorders (97). Along with addressing the urgent need for wound care, this technique consists of long-term approaches that reduce recurrence and enhance patient outcomes. To repair the underlying metabolic abnormalities that impede healing, nutritional support must accompany off-loading techniques, infection control, and revascularization (97). Furthermore, educating patients on the importance of nutrition for wound healing and foot care will encourage them to take control of their own healing and reduce complications (124).

Another key factor that impact wound outcome in diabetic patients is microcirculatory impairment. According to Valentini et al., acupuncture therapy may enhance microcirculation in patients suffering from peripheral artery disease (PAD) and diabetic foot syndrome (DFS) (125). As per their pilot research, enhancement of microvascular flow can aid in the healing of chronic wounds, which are often resistant to conventional treatment due to a lack of blood flow. This shows how important circulation-related efforts are aimed at reducing nutrient delivery deficits in diabetic wounds. Studies have analyzed the role of fungal communities in the wound microbiome in delayed healing. According to Kalan et al., the fungal population (especially Ascomycota) was found in patients as early as 8 weeks of healing (126). According to their findings, the pathophysiology of chronic wounds and related complications may be significantly influenced by fungal contributions, which are frequently disregarded in microbiome studies that concentrate on bacteria.

Moisture management remains a cornerstone of effective wound care. A study demonstrated that Acticoat Moisture Control dressings significantly accelerated closure of diabetic foot ulcers by sustaining an optimal moist wound environment, underscoring the critical role of moisture balance in promoting re-epithelialization and granulation tissue formation (127). Complementary work has shown that *Aloe vera* gel, applied topically, maintains a physiologically appropriate moisture milieu within diabetic wounds, lending credence to the concept that naturally derived polymers can stabilize the microenvironment required for efficient tissue repair (128). To dissect the biochemical derangements imposed by chronic hyperglycaemia, a three-dimensional, tissue-engineered wound model has been engineered that faithfully recapitulates the accumulation of AGEs and the attendant impairment of fibroblast proliferation, angiogenic signaling, and extracellular-matrix turnover that underlie the stalled diabetic repair program (129). Their model highlights the need for strategies that counteract these modifications by highlighting the detrimental effect of glycation on tissue regeneration. Emerging therapeutic approaches, including tissue engineering and pharmacological interventions, are being explored to improve healing outcomes. Recent comprehensive analyses have underscored the accelerating impact of bioengineered tissue scaffolds and living skin substitutes on diabetic wound closure, highlighting their capacity to reconstitute extracellular-matrix architecture, deliver pro-regenerative cytokines, and re-establish vascular networks—advances that exemplify the translational promise of regenerative medicine in resolving chronic hyperglycaemic tissue injury (130). Preliminary clinical investigations reveal that the GLP-1 receptor agonist semaglutide accelerates diabetic foot ulcer closure independently of glycaemic improvement, implying that incretin-based therapies may modulate inflammatory resolution, angiogenesis, and extracellular-matrix remodeling—thereby extending the therapeutic footprint of antidiabetic agents into the realm of tissue repair (131).

Technological developments in wound care, like instillation therapy and negative-pressure wound therapy (NPWT), have demonstrated effectiveness in accelerating wound healing in patients with diabetes (132). These treatments function by increasing granulation tissue formation, decreasing bacterial load, and improving microcirculation—all of which are essential for the healing of chronic wounds (132). However, the effectiveness of even the most cutting-edge wound care technologies can be compromised by deficiencies in essential nutrients, so treating the patient’s nutritional status is necessary for these therapies to be successful (123). A comprehensive strategy for treating diabetic foot ulcers and enhancing patient outcomes is to combine nutritional therapy with cutting-edge wound care treatments.

### 3.4 Sarcopenia and functional impairment

Sarcopenia and T2DM are becoming more widely acknowledged as linked disorders that have a substantial impact on quality of life and functional disability, especially in older populations. The progressive loss of skeletal muscle mass, strength, and function that characterizes sarcopenia is made worse by the metabolic dysregulation that is a part of type 2 diabetes. This leads to a reciprocal connection that speeds up functional deterioration (133). Compared to the general population, T2DM patients have a significantly higher prevalence of sarcopenia; studies have shown that rates vary from 20.6 to 51.9% based on age and diagnostic criteria (134). Insulin resistance, persistent hyperglycemia, and the buildup of AGEs, which hinder muscle protein synthesis and encourage muscle atrophy, are some of the pathophysiological mechanisms linked to this increased risk (135). In T2DM, impaired insulin signaling disrupts these processes, leading to reduced muscle mass and strength (136). Chronic hyperglycemia also exacerbates muscle deterioration by causing inflammation and oxidative stress. Pro-inflammatory cytokines include TNF-*α* and IL-6 that aggravate the health of muscles. AGEs accumulate in the muscle tissue, causing stiffness and impairing muscle functionality (137). T2DM patients quickly lose muscle mass and muscle function, especially in the lower body, which is essential for mobility balance due to all these reasons (138). Older individuals with type 2 diabetes who have sarcopenia suffer from decreased quality of life, increased fall risk and reduced physical performance. Research indicates that this population often has reduced muscle mass in the lower extremities which slows gait speed and decreases mobility (139). According to the Asian Working Group for Sarcopenia’s (AWGS) 2019 definition of sarcopenia, their recommendation was to utilize grip strength, gait speed and skeletal muscle mass index (SMI) to make the diagnosis. All three are significantly impaired in patients with type 2 diabetes (140). Poor control of blood sugar causes sarcopenia and functional decline, as shown by higher HbA1c levels. Efficient management of glycemic levels is required to avoid these effects (141). One cause of sarcopenia in type 2 diabetes patients is poor diet and lack of exercise which we can change. Low Mini Nutritional Assessment (MNA) scores are linked to greater muscle loss and functional disability, making malnutrition a significant contributor to sarcopenia (142).

## 4 Nutritional interventions in rehabilitation and nursing care

**Figure 2:**
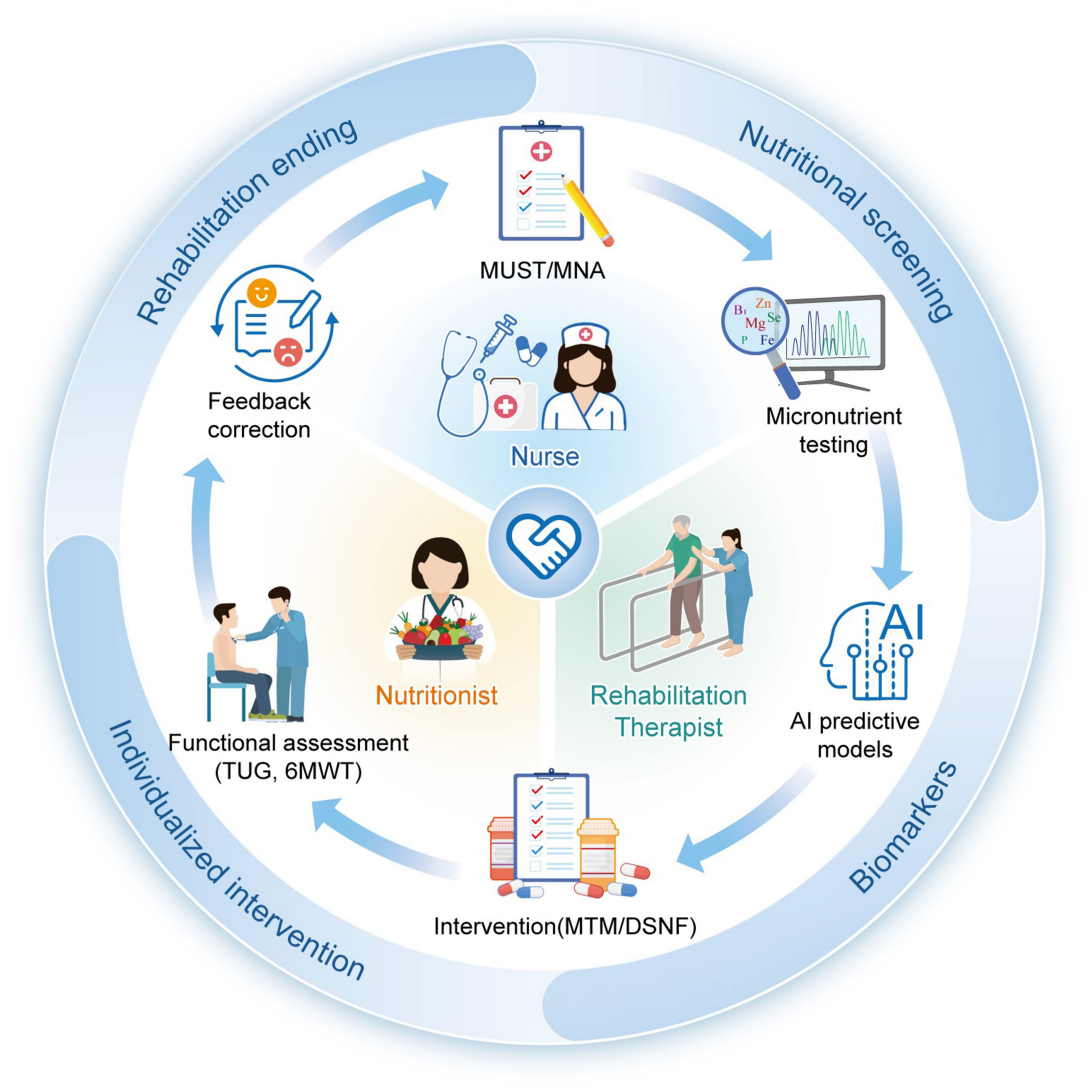
Rehabilitation nutrition care process.

Nutritional intervention is essential in nursing care and rehabilitation of an individual with T2DM because it deals with the psychological as well as physiological aspects of the disease. A component of multimodal care of type 2 diabetes, a chronic disease diagnosed with insulin resistance and hyperglycemia, is nutrition therapy (22, 143). Customized nutritional approaches have been shown to significantly improve patients’ glycemic control, metabolic outcomes, and general quality of life when incorporated into nursing care and rehabilitation programs (144, 145) (Figure 2).

Medically tailored meals (MTMs), which give patients nutrient-dense, balanced meals that are tailored to their individual dietary requirements, are among the most successful nutritional interventions. MTMs are effective for food insecure type 2 diabetes patients as they provide access to an adequate source of nutrition. As a result, healthcare utilization and costs are decreased (146). This intervention is particularly useful in rehabilitation settings where patients often have difficulty following dietary recommendations as a result of either physical complications or limited access to food. Healthcare providers can help overcome these barriers and facilitate improved disease management by incorporating MTMs into treatment plans (146).

Diabetes-specific nutrition formulations (DSNFs) are scientific formulations that have been formulated with the goal of improving glycemic control and providing essential macronutrients and micronutrients. Clinical studies have demonstrated that DSNFs, which include healthy fats, slowly digestible carbohydrates, and particular micronutrients, enhance postprandial glycemic responses and long-term glycemic control (22). These formulas can be especially helpful in nursing care settings, where patients may need nutritional support because of age-related malnutrition or comorbidities. The transcultural diabetes nutrition algorithm offers a framework for incorporating DSNFs into treatment regimens according to the requirements of each patient, including body weight and glycemic control level (22).

Programs for education and motivation are also an important part of nutritional interventions in the treatment of type 2 diabetes. Recently, numerous studies have shown that structured nutrition education improves dietary practices, metabolic parameters and self-management skills after undergoing nutrition education programs when paired with peer-to-peer (145, 147). The nine-month motivational program focusing on Mediterranean diet led to reduction in systolic and diastolic blood pressure, BMI, waist circumference, and glycemic values (144). These programs can be integrated into nursing and rehabilitation practice to provide patients with information and skills to make permanent dietary changes (144, 147).

Patients with type 2 diabetes were benefited from community-based multidisciplinary interventions that included physical activity and nutrition education. Such programs, aimed at modifying eating behavior and/or levels of physical activity, have resulted in notable improvements in glycemic control, weight status, and health-related quality of life (145). Nursing care and rehabilitation can be optimized by these programs by creating a conducive environment and imparting useful skills for self-management (145).

Micronutrient deficiencies also need to be controlled for successful management of type 2 diabetes. Many people with type 2 diabetes lack vitamin B6, vitamin C, vitamin D, and zinc. This can worsen insulin resistance, inflammation, and oxidative stress (22). Addressing those deficiencies and enhancing metabolic outcomes could be done via nutritional strategies, like the use of DSNFs or specific supplements (22). In order not to cause harm, it should be noted that supplements should be evidence-based and individualized for each patient (22). Clinical pharmacists are key players in the involvement of nutrition in type 2 diabetes therapy. Pharmacists can help manage medicines, counsel the diet, monitor metabolic parameters to help isolate clinical outcomes and patient adherence (148). By being included within interdisciplinary care teams, they are able to improve delivery of nutritional interventions and ensure that patients receive complete and well-coordinated care (148). Recent studies show how polyphenols and other bioactive compounds could improve the vascular health and metabolic outcomes of T2DM patients. The limited and conflicting evidence, on the other hand, suggests that further research in this area could lead to the development of new diets for type 2 diabetes (149). The findings stress the need for continuous research and development in the field of nutritional interventions for care nursing and rehabilitation of T2DM (149). Recent research has focused more on the investigation of new nutritional interventions in the context of nursing care and rehabilitation for people with T2DM. By including dietary tactics into all-encompassing care plans catered to the requirements of diabetic patients, especially in geriatric and rehabilitation settings, these interventions seek to maximize patient outcomes.

The importance of nutritional interventions in geriatric rehabilitation patients is highlighted by research, which shows that these tactics, whether used alone or in conjunction with physical exercise, can have a positive impact on both nutritional and functional outcomes (150). Based on this evidence, rehabilitation programs for older T2DM patients should include targeted nutritional approaches as they may improve recovery and functional independence.

Kakehi et al. emphasize the value of combined therapy further by pointing out that when nutritional therapy and exercise therapy are combined, the results are better gains in muscle strength and gait speed than when either modality is used alone (151). Because muscle preservation and functional mobility are crucial for T2DM patients with sarcopenia, this synergistic effect is especially pertinent. To address the complex needs of diabetic patients in rehabilitation settings, the findings support a multidisciplinary approach that includes both nutritional and physical interventions. Adaptable to the management of type 2 diabetes, Perry et al. offer a framework for nursing interventions targeted at enhancing nutritional status and outcomes in the larger context of nursing care (152). The parameters for effective nutritional care are outlined in their review, which also highlights the role that nurses play in putting nutritional strategies into practice and assessing them within clinical pathways. Evidence from primary-care and community-based cohorts indicates that structured, non-pharmacological interventions—centred on individualized medical nutrition therapy—constitute an indispensable pillar for achieving sustained metabolic control in T2DM (153). Comparing the efficacy of different interventions is the goal of their systematic review and planned network meta-analysis, which emphasizes the increasing acceptance of nutrition as a key component of diabetes care that goes beyond pharmacological methods.

By showing that oral nutritional supplements and wiping together can prevent aspiration pneumonia in older populations, Higashiguchi et al. provide an example of the significance of oral care as a component of nutritional intervention (154). These interventions are especially pertinent to nursing care, as preserving nutritional intake and oral health is crucial to avoiding complications in elderly patients with diabetes and frailty.

## 5 Precision nutrition and future directions

The use of lifestyle, phenotypic, and genetic information to custom-design dietary interventions that can improve glycemic control and slow down the progression of diabetes mellitus is increasingly becoming a treatment paradigm (155). This approach is extremely different to the generic dietary guidelines that are often offered in medical nutrition therapy (MNT), as these consultations rarely account for the fact that different patients respond differently to different macronutrients and other food components (156). Genetically-guided precision MNT can lead to substantial reductions in blood pressure, body weight, fasting plasma glucose, and also in glycosylated hemoglobin (HbA1c) levels, new research has found. Some patients have also been shown to reverse pre-diabetes (157). These research outcomes highlight the importance of precision nutrition and its impact on the effectiveness of dietary interventions for T2DM.

Using machine learning algorithms and continuous glucose monitoring (CGM), precision nutrition has already evolved a little further. The Twin Precision Nutrition (TPN) Program gives personalized diet recommendations based on the daily data of the Continuous Glucose Monitor (CGM) and food eaten data, enabling patients to avoid foods that spike their blood sugar and replace them with healthier ones (158). According to various studies 56.9% decrease in insulin resistance, 1.9 percentage point decrease in HbA1c and elimination of drug dependence in many individuals was observed in as little as 90 days of starting the strategy (143). These results demonstrate the revolutionary potential of integrating digital health technologies with precision nutrition to improve clinical outcomes in type 2 diabetes.

One of the key applications of ML in T2DM research is the identification of metabolomic biomarkers that can serve as early indicators of disease progression or complications. Studies have utilized ML to analyze serum metabolomic profiles, revealing significant alterations in BCAAs and gluconeogenic amino acids in T2DM patients, particularly those with complications (16). These findings highlight the potential of ML to uncover metabolic signatures that differentiate early-stage T2DM from advanced stages with complications, thereby enabling risk stratification and targeted interventions (16). ML models have been employed to predict the risk of acute coronary syndrome in T2DM patients, identifying key features such as family history of cardiovascular disease, smoking, and neutrophil count, which significantly improve predictive accuracy compared to traditional logistic regression models (159). Such applications demonstrate the clinical utility of ML in enhancing diagnostic precision and prognostic capabilities in T2DM.

The integration of ML with metabolomics has also facilitated the discovery of novel biomarkers that reflect the interplay between nutrient metabolism and T2DM pathophysiology. Targeted metabolomic analyses using ML have identified bile acids, ceramides, and amino acids as predictive biomarkers for T2DM development, while anthropometric features such as body mass index and waist circumference were found to have limited predictive value (160). This underscores the importance of metabolomic data in understanding the molecular mechanisms underlying T2DM and developing personalized intervention strategies. Additionally, tree-based ML algorithms such as XGBoost, LightGBM, and AdaBoost have been used to classify T2D patients based on metabolite data, achieving high accuracy and sensitivity in distinguishing T2D from non-diabetic controls (161). These models not only enhance diagnostic accuracy but also provide insights into the metabolic pathways implicated in T2DM, such as pyruvate metabolism and lipid metabolism, which are critical for disease progression and therapeutic targeting (161).

Another innovative application of ML in T2DM research is its use in predicting complications and poor glycemic control in nonadherent patients. Studies have demonstrated that ML algorithms can identify key risk factors for diabetic complications, such as the duration of T2D and the duration of unadjusted hypoglycemic treatment, which are crucial for improving glycemic control and reducing complication rates. By leveraging ML models, clinicians can better predict adverse outcomes in nonadherent T2D patients and implement timely interventions to mitigate risks. This approach not only improves patient outcomes but also reduces the economic burden associated with T2DM complications (162). Moreover, ML has been used to analyze gut microbiota data, revealing associations between specific microbial taxa and T2DM progression. Alterations in gut microbial members such as Alloprevotella and *Ruminococcus torques* group have been linked to elevated fasting blood glucose and glycated hemoglobin levels, highlighting the role of gut microbiota in T2DM pathogenesis (163). These findings open new avenues for developing microbiome-based diagnostic and therapeutic strategies for T2DM.

The application of ML in T2DM research also extends to the development of intelligent feature selection algorithms and optimization techniques that enhance the accuracy of predictive models. Dynamic Al-Biruni earth radius and dipper-throated optimization algorithms have been used to optimize feature selection and classification processes, achieving an accuracy of 98.6% in diabetes classification (164). Such advanced ML techniques not only improve model performance but also provide valuable insights into the most significant predictors of T2DM, such as glucose levels, body mass index, and diabetes pedigree function (164, 165). These models have the potential to be integrated into clinical practice, enabling early detection and personalized management of T2DM.

Individual responses to dietary interventions are significantly influenced by genetic variation; genome-wide association studies (GWAS) have found over 140 genomic loci associated with meal responses and T2DM predisposition (143) (Table 2). To predict a person’s vulnerability to type 2 diabetes and their chance of benefiting from particular dietary patterns, like the Mediterranean diet or low-carb diets, genetic risk scores, or GRS, have been developed (166). Glycemic characteristics and insulin sensitivity are influenced by variations in the TCF7L2 and MTNR1b genes, which have been linked to different reactions to dietary fat and carbohydrate intake, respectively (167). Through the integration of these genetic insights into precision nutrition strategies, physicians can create highly customized dietary regimens that optimize therapeutic outcomes and reduce side effects.

**Table 2:** Diet interactions increase the risk of diabetes.

Genes	Polymorphisms	Alleles	Diet interaction	Refs.
IRS1	rs2943641	T	Carbohydrate and fat intakes interact with the T allele to influence type 2 diabetes risk in a sex-specific manner. Low carbohydrate intake is associated with decreased risk in women with the T allele, while low fat intake is associated with decreased risk in men with the T allele.	(195)
ACE	rs4343 (surrogate for I/D polymorphism)	G	High fat diet intake modulates the effect of the G allele on glucose tolerance and type 2 diabetes risk. GG-carriers show significantly impaired glucose tolerance and higher type 2 diabetes risk with high dietary fat intake.	(196)
PGC-1α	rs10517030, rs10517032, rs10212638	Minor alleles	Low energy intake interacts with the minor alleles to increase type 2 diabetes risk and insulin resistance. The minor alleles are associated with higher fasting blood glucose and HOMA-IR, and lower HOMA-B in low-energy intake groups.	(197)
TCF7L2, GIPR, KCNQ1, WFS1	rs12255372, rs7903146, rs10423928, rs10010131, rs151290, rs2237892, rs163184	T (rs12255372)	Coffee intake interacts with the T allele in TCF7L2 to influence type 2 diabetes risk. Carriers of the T allele benefit more from coffee consumption in terms of reduced type 2 diabetes risk.	(198)
Adiponectin	SNP276G>T	G, T	Carbohydrate intake interacts with the SNP276G > T polymorphism to affect fasting blood glucose, HbA1C, and HDL cholesterol. The genetic effect on these parameters depends on the percentage of total energy intake from carbohydrates.	(199)
FTO, MC4R	rs9939609 (FTO), rs17782313 (MC4R)	A (FTO), C (MC4R)	Adherence to a Mediterranean dietary pattern attenuates the association between FTO and MC4R polymorphisms and type 2 diabetes risk; conversely, among individuals with low Mediterranean-diet adherence, carriers of the FTO and MC4R risk alleles exhibit a significantly higher risk of type 2 diabetes.	(200)
TCF7L2	rs7903146	T	High dietary fiber intake may amplify the association between the TCF7L2 rs7903146 T allele and type 2 diabetes; among high-fiber consumers, the T allele is linked to elevated HbA1c levels, whereas this association is attenuated in low-fiber consumers.	(201)
CF7L2	rs7903146	T	In the Algerian population, high dessert intake amplifies the association between the TCF7L2 rs7903146 T allele and type 2 diabetes: T-allele carriers with high dessert consumption exhibit a significantly higher risk of type 2 diabetes and elevated fasting plasma glucose levels.	(202)
IL-6	rs1800795, rs1800796	C (rs1800795), C (rs1800796)	High sugar intake and physical activity status significantly influence IL-6 levels, although their associations with type 2 diabetes risk remain equivocal.	(203)
IL-6	rs1800795	C, G	The IL-6 rs1800795 polymorphism exhibits a protective effect against T2DM in Asian and mixed-ancestry populations but is associated with an increased risk of T1DM.	(204)
PPARG	rs1801282	Ala12	Interactions between unsaturated fatty acids and PPARG variants may modulate body weight and glucose homeostasis.	(205)
SLC30A8	rs13266634	T	Zinc intake interacts with SLC30A8 variants to potentially modulate glycaemic levels.	(206)
CDKN2A/B	rs10811661	A	An interaction between low birth weight and CDKN2A/B risk alleles increases susceptibility to type 2 diabetes.	(207)
GIPR	rs10423928	A	A high-fat diet attenuates type 2 diabetes risk via interaction with GIPR, whereas a high-carbohydrate diet elevates this risk through the same genetic interaction.	(208)
GIPR	rs10423928	T	A high-carbohydrate diet interacts with the T allele to reduce type 2 diabetes risk, whereas a high-fat diet synergizes with the T allele to increase this risk.	(208)
HHEX, JAZF1	rs1111875(HHEX), rs864745(JAZF1)	G	Low birth weight synergizes with HHEX and JAZF1 risk alleles to increase type 2 diabetes susceptibility.	(207)

AHEI, Alternate Healthy Eating Index; DASH, Dietary Approaches to Stop Hypertension; G × D interactions: gene–diet interactions; GWAS, genome-wide association study; IDI, inflammatory diet index; MDS, Mediterranean diet score; PRS, polygenic risk score; RCT, randomized controlled trial; SNPs, single nucleotide polymorphisms; GIPR, glucose-dependent insulinotropic polypeptide receptor gene.

Intermittent fasting (IF) has emerged as a promising intervention in the field of precision nutrition. In particular, IF’s potential to improve glucose metabolism and enhance insulin sensitivity (138). To identify the patient subgroups that are most likely to benefit from IF, recommendation systems based on machine learning have been developed. These systems take into account variables such as age, gender and BMI (139). Individuals can undertake personalized strategies for intermittent fasting (IF) to find out which can help them the most. Moreover, this will be possible because of the three systems. In addition, the use of IF in conjunction with other precision nutrition strategies, such as genetically-guided MNT, would provide a comprehensive strategy to manage the complex pathophysiology of type 2 diabetes.

A significant topic of interest in this field is the impact of gut microbiota on the reactions to dietary intake and disease. According to Hughes et al., in the study of interindividual variability in dietary responses, complex data which includes microbiome just cannot (168). According to their subsequent study, the gut microbiota highly influences dietary responses in each individual. Additionally, models that rely on microbiomes may enhance the effectiveness of dietary treatments proposed to combat type 2 diabetes (168).

Precision nutrition approaches that incorporate microbiome data are thought to be a promising way to enhance individual results. Developing customized treatments for type 2 diabetes requires an understanding of the microbiome’s role in metabolic regulation and its capacity to forecast how the body will react to dietary changes (168). This is in line with larger initiatives to improve dietary recommendations based on individual microbiota compositions by integrating multi-omics data into clinical practice. Beyond microbiome considerations, multidisciplinary research initiatives also influence the direction of T2DM precision nutrition. In their discussion of current military nutrition research, Karl et al. highlight operational needs, dietary habits, and methods for maximizing nutrient delivery—principles that can be applied to customized nutrition frameworks for long-term conditions (169). Their focus on creating flexible nutrient delivery plans emphasizes how crucial customized strategies are for treating complicated metabolic disorders. The relationship between nutrition, microbiota, and illness management is also being investigated by new research. Greathouse et al. examine the potential and difficulties of using precision nutrition to treat cancer, emphasizing the vital role that interactions between the diet and microbiota play (170). Despite being oncology-focused, their understanding of how dietary patterns affect the composition of the microbiome and treatment outcomes is applicable to type 2 diabetes, where microbiome modulation may enhance metabolic control and treatment effectiveness. Precision nutrition is being advanced in large part by technological advancements. An extensive review of artificial intelligence (AI) applications in personalized nutrition is given by Agrawal et al., who highlight how AI-driven models can use real-time biological data to produce customized dietary recommendations (171). It is anticipated that these developments will convert static dietary recommendations into flexible, data-driven frameworks that can accommodate the variation seen in T2DM patients.

Apart from technological advancements, the incorporation of molecular tools like small interfering RNAs (siRNAs) and microRNAs (miRNAs) presents encouraging opportunities for tailored management of type 2 diabetes and obesity. According to Humayrah et al., these molecular regulators have the ability to alter the expression of genes linked to metabolic pathways, allowing for more individualized dietary interventions based on each person’s unique genetic and epigenetic characteristics (172).

The widespread application of precision nutrition for type 2 diabetes still faces obstacles in spite of these developments. For some populations, the availability of these interventions may be restricted by the high expense of genetic testing and cutting-edge technologies combined with inequalities in healthcare access (140). Precision nutrition adoption in clinical practice is significantly hampered by the absence of standardized guidelines and the requirement for additional research to confirm its long-term efficacy and safety (173). Researchers, clinicians, legislators, and industry stakeholders must work together to address these issues and make precision nutrition available, economical, and efficient for all people with type 2 diabetes.

## 6 Conclusion

Precision nutrition is a revolutionary approach that is being used for the treatment of type 2 diabetes. Integrating precision nutrition into the management of type 2 diabetes through a multidisciplinary approach can enhance glycemic control, reduce complications, and improve patient rehabilitation and quality of life. More efficient, individualized, and long-lasting treatment of this chronic metabolic disease will be possible by addressing the intricate interactions between genetic, phenotypic, and lifestyle factors.

## Glossary

T2DM: Type 2 diabetes mellitus
DPN: Diabetic peripheral neuropathy
DKD: Diabetic kidney disease
DFUs: Diabetic foot ulcers
SGLT2: Sodium-glucose cotransporter-2
GLP-1: Glucagon-like peptide-1
NDT: Neurodynamic techniques
NM: Neural mobilization
DTI: Diffusion tensor imaging
ADC: Apparent diffusion coefficient
FA: Fractional anisotropy
NAFLD: Nonalcoholic fatty liver disease
CKD: Chronic kidney disease
MNT: Medical nutrition therapy
GRS: Genetic risk score
GWAS: Genome-wide association studies
IF: Intermittent fasting
TPN: Twin precision nutrition
CGM: Continuous glucose monitoring
HbA1c: lycosylated hemoglobin
TCA: Tricarboxylic acid
BCAAs: Branched-chain amino acids
AGEs: Advanced glycation end-products
Nnmt: Nicotinamide N-methyltransferase
ERK: Extracellular signal-regulated kinase
HNFs: Hepatocyte nuclear factors
HKDC1: Hexokinase domain-containing protein-1
UK-EDM: United Kingdom Early Diabetes Mellitus
UKPDS: United Kingdom Prospective Diabetes Study
IDF: International Diabetes Federation
ADA: American Diabetes Association
EASD: European Association for the Study of Diabetes
JDRF: Juvenile Diabetes Research Foundation
NICE: National Institute for Health and Care Excellence
WHO: World Health Organization
FDA: Food and Drug Administration
CDC: Centers for Disease Control and Prevention
NIH: National Institutes of Health
AHA: American Heart Association
